# The Adult Human Brain Harbors Multipotent Perivascular Mesenchymal Stem Cells

**DOI:** 10.1371/journal.pone.0035577

**Published:** 2012-04-16

**Authors:** Gesine Paul, Ilknur Özen, Nicolaj S. Christophersen, Thomas Reinbothe, Johan Bengzon, Edward Visse, Katarina Jansson, Karin Dannaeus, Catarina Henriques-Oliveira, Laurent Roybon, Sergey V. Anisimov, Erik Renström, Mikael Svensson, Anders Haegerstrand, Patrik Brundin

**Affiliations:** 1 Neuronal Survival Unit, Department of Experimental Medical Science, Wallenberg Neuroscience Center, Lund University, Lund, Sweden; 2 Department of Neurology, Scania University Hospital, Lund, Sweden; 3 Lund University Diabetes Centre, Scania University Hospital, Malmö, Sweden; 4 Department of Neurosurgery, Scania University Hospital and Lund Stem Cell Center, Lund University, Lund, Sweden; 5 The Rausing Laboratory, Department of Clinical Medicine, Lund University, Lund, Sweden; 6 NeuroNova AB, Stockholm, Sweden; 7 Department of Neurosurgery, Karolinska Hospital, Stockholm, Sweden; 8 Translational Neurology Group, Department of Clinical Sciences, Wallenberg Neuroscience Center, Lund University, Lund, Sweden; 9 Center for Neurodegenerative Science, Van Andel Research Institute, Grand Rapids, Michigan, United States of America; Seattle Children's Research Institute, United States of America

## Abstract

Blood vessels and adjacent cells form perivascular stem cell niches in adult tissues. In this perivascular niche, a stem cell with mesenchymal characteristics was recently identified in some adult somatic tissues. These cells are pericytes that line the microvasculature, express mesenchymal markers and differentiate into mesodermal lineages but might even have the capacity to generate tissue-specific cell types. Here, we isolated, purified and characterized a previously unrecognized progenitor population from two different regions in the adult human brain, the ventricular wall and the neocortex. We show that these cells co-express markers for mesenchymal stem cells and pericytes *in vivo* and *in vitro*, but do not express glial, neuronal progenitor, hematopoietic, endothelial or microglial markers in their native state. Furthermore, we demonstrate at a clonal level that these progenitors have true multilineage potential towards both, the mesodermal and neuroectodermal phenotype. They can be epigenetically induced *in vitro* into adipocytes, chondroblasts and osteoblasts but also into glial cells and immature neurons. This progenitor population exhibits long-term proliferation, karyotype stability and retention of phenotype and multipotency following extensive propagation. Thus, we provide evidence that the vascular niche in the adult human brain harbors a novel progenitor with multilineage capacity that appears to represent mesenchymal stem cells and is different from any previously described human neural stem cell. Future studies will elucidate whether these cells may play a role for disease or may represent a reservoir that can be exploited in efforts to repair the diseased human brain.

## Introduction

Mesenchymal stem cells (MSC) are the conceptual postnatal progenitors of most derivatives of mesoderm [1], [2]. They were initially isolated from the bone marrow [3], but subsequently also from several other tissues e.g. the umbilical cord, bone trabeculae, muscle, synovium, dental pulp, periodontal ligament and adipose tissue [1], [4]. Mesenchymal stem cells are isolated by adherence to plastic, and characterized by the expression of a panel of surface markers [5] and their capacity to differentiate along mesodermal lineages into adipocytes, chondroblasts and osteoblasts [3]. Until recently, the exact *in vivo* identity of MSC was elusive. However, now it has been suggested that MSC may reside in the perivascular compartment and have characteristics identical to a subclass of pericytes [4], [6], [7], [8]. Pericytes reside on the abluminal surface of endothelial cells in the perivascular space and span the entire microvasculature. Not only are they important regulators of angiogenesis and blood vessel function [9] they also contribute to the pathogenesis of diabetic microangiopathy, cancer, atherosclerosis and Alzheimer's disease [10]. Akin to MSC, pericytes have been reported to be able to differentiate into osteoblasts [11], [12], chondrocytes and adipocytes [13], [14]. Observations in several tissues suggest that they can contribute to tissue repair: pericytes differentiate into adipocytes during fat tissue injury [15], into chondroblasts and bone after bone injury [12], into myoblasts in a model for muscular dystrophy [16] and into Leydig cells of the testis [17]. Recent data in a mouse model show that pericytes have the ability to contribute to spinal cord repair by differentiation into astrocytes [18]. Interestingly, the highest density of pericytes is found in the central nervous system [19] and it is not known whether the human perivascular compartment harbors this particular subclass of pericytes and whether this cell type has stem cell properties.

Here, for the first time, we identify a perivascular stem cell in the human adult brain. We isolate, purify and characterize cells from human brain biopsies that resemble marker expression of the perivascular progenitors found in vivo. We show that these cells share a mesenchymal and pericyte phenotype and have the potential to differentiate into mesodermal and neuroectodermal progeny.

## Results

### The adult human brain contains cells that co-express mesenchymal and pericyte markers

**Figure 1:**
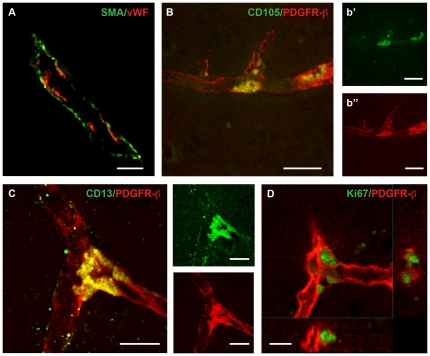
The adult human brain contains perivascular cells co-expressing mesenchymal stem cell and pericyte markers. (A) Confocal image showing neocortical brain section with intracerebral capillary with endothelial cells on the luminal side of the blood vessel (vWF, red) surrounded by α-SMA-positive cells (green). Scale bar 100 µm. (B) Capillaries are lined with pericytes expressing PDGFR-β/CD140b (red) whereby a proportion double labeled for the MSC markers CD 105 (green) and (C) CD13 (green). Scale bar 25 µm (B) and 17 µm (C). (D) PDGFR-β-positive cells at vessel branching points stain for Ki67 (green). Scale bar 10 µm.

We examined sections of the human neocortex for the presence of cells expressing MSC markers. Cells positive for α-smooth muscle antigen (α-SMA), a marker for smooth muscle cells and pericytes [20] lined microcapillaries (Figure 1A). We identified cells expressing the pericyte marker platelet-derived growth factor receptor β (PDGFR-β) along the perivascular space [21], [22]. A subpopulation of PDGFR-β-positive pericytes co-expressed markers for MSC (CD105 and CD13) and was preferably situated at vessel branching points (Figure 1B, C). The PDGFR-β-positive pericytes located at the branching point of vessels also expressed Ki67, a marker associated with cell proliferation (Figure 1D). Interestingly, also the cells labeling for MSC markers were found at branching points, suggesting that this is a proliferating population. We therefore refer to these cells as perivascular MSC hereafter.

### Isolation, sorting and expansion of human mesenchymal progenitor cells

**Figure 2:**
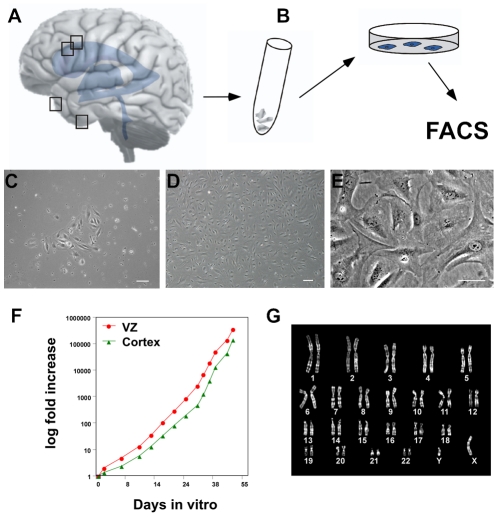
Isolation and expansion of perivascular mesenchymal stem cells from the adult human brain. (A) Progenitor cells were isolated from fresh tissue biopsies from the ventricular wall (n = 2) or the temporal neocortex (n = 2). (B) The tissue was enzymatically digested and cells were plated and expanded as adherent monolayers. Once sufficient cell numbers were reached cells were purified by FACS. (C) Phase contrast image of morphology of adherent growing progenitor cells 5 days after isolation from neocortical tissue and (D) at confluency. (E) Cells have the typical flat morphology and large nucleus with prominent nucleoli as described for perivascular cells. Scale bars 50, 10, 100 µm. (F) Exponential growth characteristics of cell lines from ventricular zone and the cortex. Progenitors exhibit similar doubling time independent of region of origin. (G) Karyotype at passage 39, showing euploid number of chromosomes following long-term expansion. Data from representative culture from ventricular zone donor line.

Fresh tissue biopsies from the ventricular zone or the temporal neocortex of the human brain were carefully enzymatically dissociated, plated on plastic dishes and subjected to Fluorescence-activated cell sorting (FACS) once high enough cell numbers had been obtained (Figure 2A, B). Within a few days after plating the first proliferating adherent cells were visible (Figure 2C). Only very few primary neural cells or cell aggregates were visible in freshly plated cultures but were lost when the cell cultures had been passaged twice. Passaging and replating on uncoated plastic dishes selected for the adherent cell type (Figure 2D, E).

Cell lines from both the ventricular zone and the cortex exhibited similar logarithmic growth expansion (Figure 2F) and could be freeze-stored and thawed without losing their proliferation capacity. We were able to efficiently long-term propagate cells over many passages (up to passage 50 assessed so far). Long-term expanded cells kept a normal karyotype (Figure 2G).

**Figure 3:**
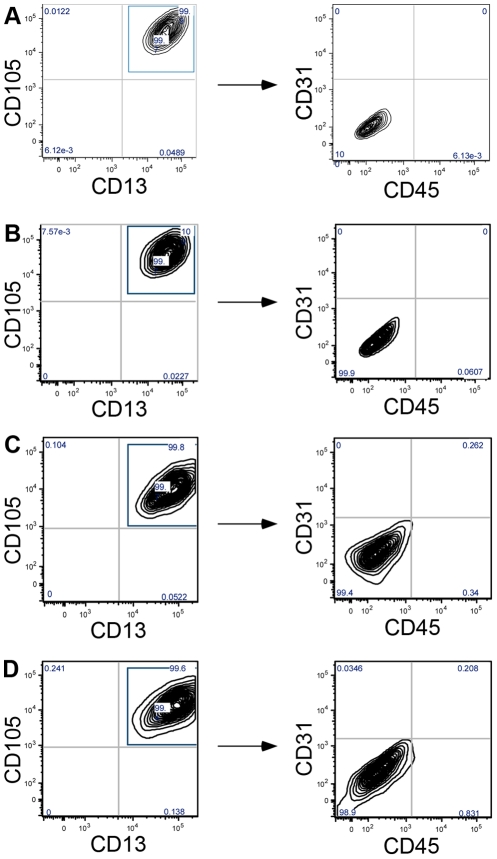
FACS sorting of brain-derived progenitor cell lines. Human progenitor cells were positively sorted for the MSC markers CD105 and CD13, and thereafter negatively for hematopoietic (CD45) and endothelial markers (CD31). Histogram illustrates results (A, B) for the two different cortical lines and (C, D) for the two lines from the ventricular zone. 99.4–100% of the cell population expressed both, CD105 and CD13, and cultures did not contain endothelial or hematopoietic cells.

We used FACS to purify the cells. We sorted cell lines from all donors by first positively gating for MSC markers CD13 and CD105; and then negatively for the hematopoietic marker CD45 and the endothelial marker CD31. The CD13^+^CD105^+^CD45^−^CD31^−^ population comprised 99.4–100% of the isolated and propagated cell populations irrespective of which donor they originated from and whether they were derived from the ventricular zone or the neocortex (Figure 3A–D). Based on the marker expression found in vitro we concluded that the isolated cells resemble the perivascular MSC in vivo.

### Co-expression of mesenchymal and pericyte markers

**Figure 4:**
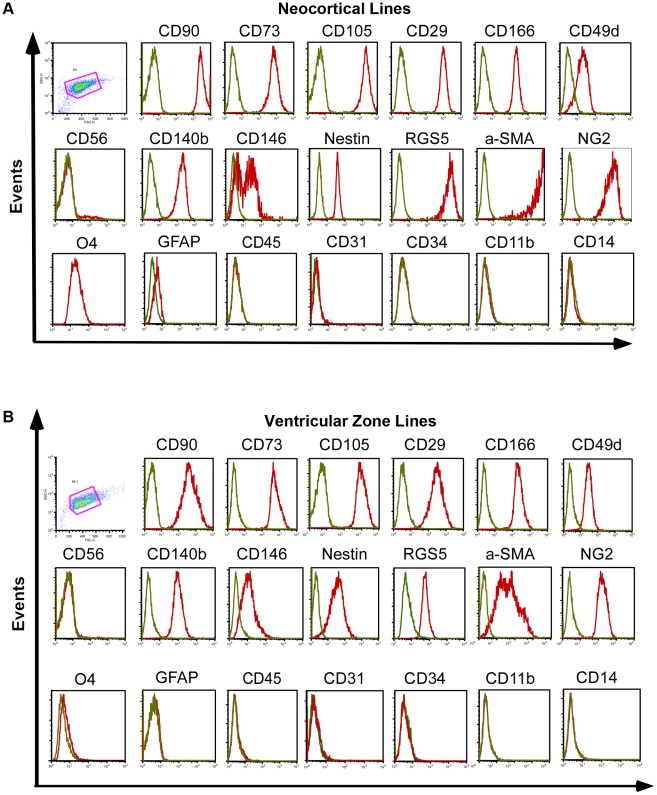
Brain-derived cell lines express markers for mesenchymal stem cells and pericytes but not for glial or neuronal precursors. (A) Representative histogram of flow cytometry analysis of cortical line and (B) ventricular zone line. Progenitors from all donors and both regions highly express MSC (CD90, CD73, CD105, CD29, CD166 and CD49d) and pericyte markers (CD140b/PDGFR-β, RGS5, CD146, Nestin, α-SMA and NG2). They do not express hematopoietic (CD45), endothelial (CD31, CD34), microglial (CD14, CD11), glial or neuronal precursor cell markers (GFAP, O4) or myofibroblast markers (CD56), (green = isotype, red = respective marker).

We further investigated the antigenic profile of the cell lines using flow cytometry analysis. Cell lines from all donors (n = 4) expressed several MSC markers, such as CD105 (98.98% ±0.5), CD73 (96.0% ±2.9), CD90 (88.5% ±5.2), CD29 (99.4% ±0.2), CD166 (96.0% ±2.4) and CD49d (89.2% ±4.1) but not the myoblast marker CD56 (4.4% ±0.8) (Fig. 4A and B). Furthermore, the cell lines expressed a panel of phenotypic markers currently used to identify pericytes [8], [16] such as PDGFR-β/CD140b [21], [22] (97.8% ±0.7), CD146 [8] (57.3% ±7.3), regulator of G-protein signaling 5 (RGS5), a marker selectively expressed in pericytes in brain capillaries [23] (81.7% ±12.1), Nestin [24] (94.1% ±2.7), α-SMA [20] (96.6% ±1.2) and high molecular weight melanoma-associated antigen NG2 [25] (78.3% ±21.5). Cells derived from the four different donors neither expressed glial precursor markers such as O4 (1.4% ±1.0) and glial fibrillary acid protein (GFAP, 3.1% ±0.9) nor hematopoietic (CD45, 1.4% ±0.5), endothelial (CD31, 1.3% ±0.4; CD34, 1.4% ±0.1) or microglial markers (CD11b, 1.1% ±0.2; CD14, 0.9% ±0.2) (Fig. 4A, B).

**Figure 5:**
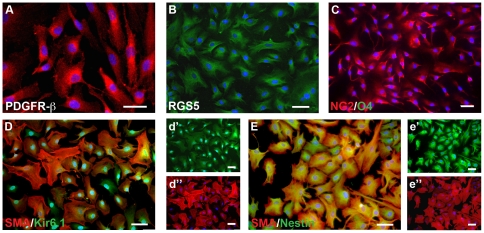
Progenitor cells exhibit a pericyte phenotype in vitro. (A) Immunofluorescence image showing expression of PDGFR-β/CD140b, (B) the pericyte-specific marker RGS5 and (C) expression of NG2 but absence of the oligodendroglial marker O4. Cells also co-express (D) Kir6.1, a marker specific for cerebral pericytes (d′) and α-SMA (d″, e″), and (E) Nestin (e′) as previously described for pericytes. Nuclei are stained with DAPI. Scale bars 50 µm.

Consistent with the flow cytometry analysis, using immunocytochemistry we confirmed that cells expressed the pericyte markers PDGFR-β/CD140b, RGS5, NG2 but not the glial precursor marker O4. Cells co-labeled with α-SMA, Nestin and Kir6.1, a marker specific for cerebral pericytes [26]. Results did not differ between donors or region of origin (ventricular zone or neocortex). Representative images from a ventricular donor line are presented (Figure 5A–E). Furthermore, proliferating cell lines did not stain for the neural or glial precursor cell markers GLAST (astrocyte-specific glutamate transporter), GFAP, S-100β or A2B5 (data not shown).

### Characteristics of clonally derived progenitors

**Figure 6:**
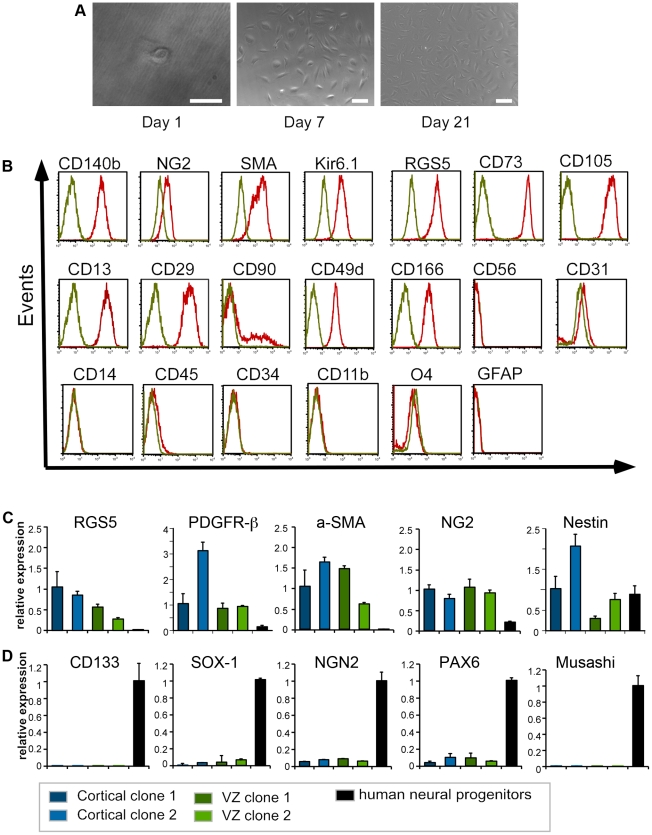
Characterisation of clonal perivascular MSC progenitor lines. (A) Single cell *in vitro* a few hours after plating, and culture at 7 and 21 days, respectively. Scale bar 50 µm. (B) Clonally derived MSC show the same antigen profile as detected for polyclonal lines with pericyte and MSC markers but neither neuronal nor glial precursor cell markers (O4, GFAP) (example here cortical clone; green = isotype, red = respective marker).(C) QPCR comparing clones derived from the ventricular zone (n = 2), and the cortex (n = 2) to a human neuronal progenitor cell line MesC2.10. Data show the relative higher expression of mRNA for RGS5, PDGFR-β and α-SMA in the MSC clones compared to the control. As expected, Nestin mRNA was present in perivascular MSC clones and neural progenitors at comparable amounts. NG2 mRNA was slightly higher expressed in the MSC clones compared to the control. (D) Importantly, neural progenitors markers such as CD133, SOX1, NGN2, PAX6 and Musashi were highly expressed in the positive control but not detectable in MSC clones.

Next, we derived clones from all cell lines to investigate differentiation properties of the brain-derived perivascular MSC (Figure 6A). Single cell-derived clones were derived from all donors with minimal variation in cloning efficacy (1–3%). Clones had the same antigenic profile as FACS-sorted polyclonal cells (Figure 6B). As cells were derived from the human brain, expanded monoclonal cell lines from all donors were also examined for the presence of mRNA for neural progenitor markers to exclude that the population was derived from a neural progenitor. The human neuronal progenitor line MesC2.10 [27] served as a positive control. Single-cell derived clones expressed high levels of mRNA for pericyte markers RGS5, PDGFR-β and α-SMA compared to the human neuronal progenitors. As expected, clonal lines derived from perivascular MSC and the control neuronal progenitors expressed comparable levels for Nestin and NG2 mRNA (Figure 6C). Both, Nestin and NG2 are markers that are described for neuronal progenitors, but are also well-recognized markers for pericytes [24], [25], [28] which explains this overlap in marker mRNA. However, most importantly, mRNA for the neuronal progenitor markers CD133, SOX1, NGN2, PAX6 and Musashi was not detectable in any of the monoclonal lines, but highly expressed in the control neuronal progenitor line (Figure 6D).

### Clonally derived perivascular MSC differentiate along the mesodermal lineage

**Figure 7:**
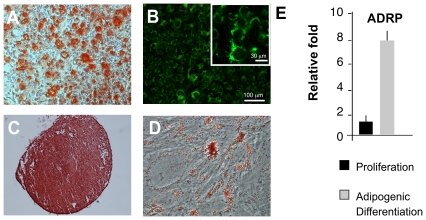
Human brain-derived clonal MSC have mesodermal potential. Clones derived from both, the ventricular zone (n = 4) and the cortex (n = 6) can be induced to give rise to formation of osteoblasts; (A) Alzarin S staining and (B) osteocalcin staining when cultured in osteogenic medium for 21 days. (C) The same clone forms chondrocytes if cultured as pellets (450 000 cells/pellet) in induction medium. Image shows Safranin O Red staining of cortical-derived clone. (D) Culture in adipogenic medium leads to lipid-containing adipocytes after 14 days. Staining with Oil red illustrates accumulation of typical lipids. (E) qPCR shows about 8-fold increase of adipose differentiation related protein (ADRP) in cultures exposed to adipogenic differentiation medium after 14 days compared to non-induced controls.

The fact that the brain-derived cells expressed MSC and pericyte markers prompted us to investigate their mesodermal potential. Using previously described protocols [8] we successfully induced clonal lines from both the neocortex (n = 6) and the ventricular zone (n = 4) to differentiate into osteoblasts, chondrocytes and adipocytes as evidenced by stainings with the typical markers (Figure 7A–D). Control cultures in proliferation medium did not stain for any of the markers (data not shown). Furthermore, we observed a 8-fold upregulation of mRNA for the adipose differentiation related protein (ADRP) in perivascular MSC's cultured in adipogenic medium compared to non-induced control cultures (Figure 7E).

### Clonally derived perivascular MSC differentiate along the neuroectodermal lineage

Pericytes in different organs share a similar marker expression profile. However, the tissue they reside in may influence their differentiation capacity [7]. Therefore, we investigated if human cerebral perivascular MSC can adopt the phenotype of cells found in the brain.

**Figure 8:**
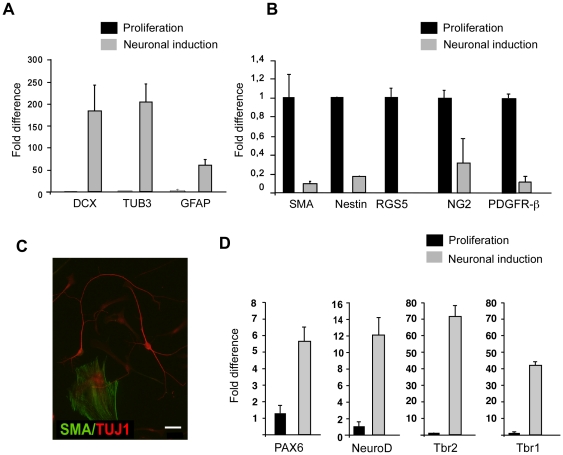
Human brain-derived clonal MSC upregulate immature neuronal markers and downregulate pericyte markers upon neuronal induction. QPCR in neural induction cultures. (A) After 10 days of neuronal induction, we observed a 180–200 fold upregulation of mRNA of the early neuronal markers DCX and β-III-tubulin and an about 50-fold increase in mRNA for GFAP, a glial marker and marker for neural progenitors. (B) At the same time, mRNA expression for pericyte markers Nestin, α-SMA, RGS5 and PDGFR-β decreased compared to undifferentiated controls. (C) Immunofluorescence image of neuronal induction culture showing TUJ1-positive cells and α-SMA-expressing cells. (D) QPCR showing upregulation of neuronal transcription factors. There was a more than 6-fold upregulation in PAX6, tvelve-fold upregulation of NeuroD1, seventy three-fold upregulation of Tbr2 and forty two-fold upregulation of Tbr1 compared to undifferentiated cells.

When clonal cell lines were cultured using a neuronal induction protocol (see methods), mRNA for the neural markers doublecortin (DCX), β-III-tubulin and GFAP was highly upregulated compared to proliferating MSC, thus confirming the neuronal induction (Figure 8A). In proliferating, non-induced cultures no mRNA for neuronal markers was detected (Figure 8A, B). At the same time mRNA for neuronal markers was upregulated in cultures induced in neural induction medium, the expression of the pericyte markers α-SMA, Nestin, RGS5, NG2 and PDGFR-β was significantly decreased compared to proliferating controls (Figure 8B). This was consistent with the changes in protein expression seen by immunocytochemistry (Figure 8C).

We then investigated the expression of mRNA for different neuronal transcription factors. Differentiating cells showed a sixfold upregulation of the neuronal transcription factor Pax6, a twelvefold increase in mRNA for NeuroD1, and a 73-and 42-fold upregulation of Tbr2 and Tbr1, respectively, indicating a glutamatergic phenotype (Figure 8C).

**Figure 9:**
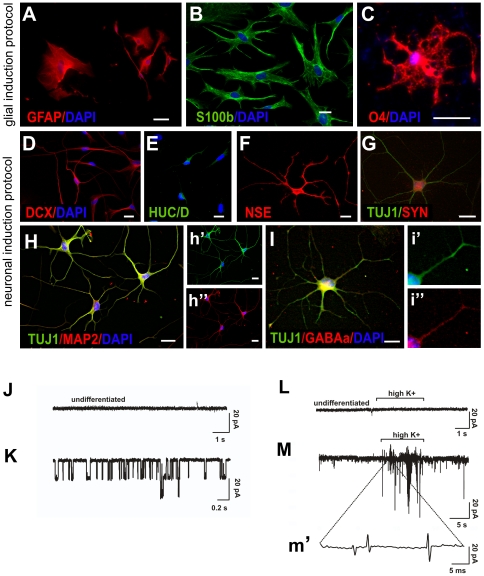
Human brain-derived clonal mesenchymal stem cells have neuroectodermal potential. (A) Image shows GFAP-staining, (B) S100β-staining and (C) O4-staining after differentiation in glial induction medium. (D) If cells were exposed to neuronal induction protocol, cells stain positive for doublecortin (DCX). Ten days after neuronal induction, differentiating cells express HUC/D (E), neuron-specific enolase (NSE) (F) and the early neuronal marker β-III-tubulin (TUJ1) (G, H, h′, I, i′). Few cells express synaptophysin (G) and the pan-neuronal marker Map2 (H, h″). A few TUJ1-positive cells express GABAA-receptor (I, i″). Scale bars 50 µm. (J) Patch clamp recording of membrane currents using the cell-attached mode, in undifferentiated control cells. Note the absence of single-channel activity. (K) In contrast, single channel activity recorded in a cell differentiated in neural induction medium for 7 days. (L) Absence of electrical responses in a depolarized control cell. (M) Action currents, reflected as the first derivative of the membrane current, recorded in a depolarized differentiated cell. Several action currents are magnified in (m′). Data shown are representative of 5 experiments in differentiated and undifferentiated cells.

Next, we used immunocytochemistry to further investigate the phenotype of differentiated cells. We used two different protocols. When clonal cultures were grown in glial induction medium (see methods) the majority of cells expressed the glial markers GFAP and S100β (Figure 9A, B) and few cells the oligodendrocyte marker O4 (Figure 9C). If exposed to neuronal induction medium (see methods) for 7 days, cells stained for the immature neuronal marker doublecortin (DCX) (Figure 9D). 10 days after neuronal induction, cells expressed HUC/D (Figure 9E), neuron specific enolase (NSE) (Figure 9F) and the immature neuronal marker β-III-tubulin (Figure 9G, H, I). A proportion of those stained positive for the pan-neuronal marker MAP2 (Figure 9H, h″). Exposure to glial induction medium resulted in 40.9% ±2.4 GFAP-positive cells and 11.4%±1.6 O4-positive cells, whereas in neuronal induction medium, 30.4% ± 4.18 of the total number of cells expressed TUJ1. The remaining cells continued to express markers for proliferating cells. Few neuronal cells also labeled with synaptophysin and PSD 95 (Figure 9G and data not shown). Only very few cells (<1%) adopted a more mature neuronal morphology and expressed GABAA-receptor (Figure 9I) but were negative for dopaminergic, serotonergic or cholinergic markers. Neuronal and glial cells could be derived from both regions, i.e. the ventricular zone (4 clones analyzed) and the neocortex (6 clones analyzed).

We then used the patch-clamp technique to examine the electrophysiological properties of clonally derived cells that displayed a neuronal phenotype in comparison to undifferentiated controls. Undifferentiated perivascular progenitor cells were electrically silent under baseline conditions and failed to produce responses to depolarizing stimuli (Figure 9J, L). In contrast, we observed single-channel activity in differentiated cells with immature neuronal morphology (Figure 9K). The mean open time of the recorded channel openings was 8.1±1.5 ms with a single channel current of 15.9 pA and a conductance of 214 pS at −70 mV. Upon stimulation with high potassium, action currents were observed in differentiated cells with neuronal morphology (Figure 9M, m′).

## Discussion

We have identified a previously uncharacterized multipotent progenitor cell in the perivascular compartment of the adult human brain. Using FACS purification, flow cytometry analysis, immunocytochemistry and qPCR we determine that an isolated progenitor cell exhibits a marker signature for both, MSC and pericytes in vitro.

Perivascular cells and MSC have previously been linked [8], [13], [29]. Recently it was suggested that the in vivo location of MSC may be the perivascular niche and, that MSC may actually represent a subclass of pericytes [6], [7]. Consistent with this notion, we identified cells expressing mesenchymal (CD105, CD13) and pericyte markers (PDGFR-β) in a perivascular location in the adult human brain, primarily around vascular branching points. This population is dividing as indicated by Ki67 staining, a proliferation marker.

We isolate, expand and positively sort the progenitors for CD105 and CD13 and negatively for the hematopoietic marker CD45 and the endothelial CD31. Thereby we show that the isolated and expanded progenitor cells resemble the marker expression of perivascular pericytes in vivo. We further demonstrate that polyclonal and monoclonal cerebral perivascular MSC are highly positive for pericyte and MSC markers but negative for hematopoietic, endothelial, microglial and glial markers. We show that clonally derived cerebral perivascular MSC can differentiate along multiple mesodermal lineages in adipocytes, chondrocytes and osteoblasts. This has not been reported for a human brain-derived progenitor cell before, but is a known feature of both MSC from other tissues [3] and pericytes [11], [12], [13], [14].

Neuroectodermal differentiation capacity of MSC has been observed in MSC from bone marrow when cells were exposed to certain epigenetic signals [30], [31], [32], [33], [34], or when MSC were genetically transduced [35]. Thus, MSC may have an inherent potential to differentiate into multiple lineages if exposed to appropriate epigenetic signals, although this capacity may differ between different tissues of origin. Interestingly, under pathological conditions, a tissue-specific differentiation capacity of pericytes has been observed. Pericytes differentiate into adipocytes during fat tissue injury, or into chondroblasts, bone, myoblasts or Leydig cells, depending on their location [12], [15], [16], [17]. Recently, pericytes have been shown to contribute to spinal cord repair by differentiation into astrocytes [18]. Our findings support previous data in primates and rodents that indicate the possible derivation of neurons from pericytes in the central nervous system. Thus, it was previously suggested that vascular adventitia may generate neural progenitors after transient ischemia in monkeys [36] and another study provided evidence in rats that isolated CNS pericytes have the potential to become cells that express neuronal markers [37].

We show using PCR and immunocytochemistry that perivascular MSC derived from the human brain not only differentiate into mesodermal lineages, but can be epigenetically induced to differentiate along glial and neuronal lineages. Perivascular MSC change morphology, downregulate pericyte markers and express mRNA for neuronal transcription factors (Pax6, NeuroD1, Tbr2, Tbr1) as well as for doublecortin and TUJ1. We also detect protein expression, though to a low degree, of more mature neuronal markers (HUC/D, NSE, Map2, synaptophysin, GABAA receptor) upon epigenetic simulation. Using a glial induction protocol, cells adopt a glial phenotype and express glial markers (GFAP, S100β, O4). Thus, human cerebral perivascular MSC have the inherent capability to generate glial cells and neuronal cells. The differentiating cells clearly show changes in membrane properties compared to proliferating cells indicating a change in phenotype even though, under the culture conditions we explored, the majority of cells have an immature neuronal phenotype and immature membrane properties. A small percentage of cells express markers resembling the phenotype of mature glutamatergic neurons, consistent with the mRNA expression of neuronal transcription factors. Further studies are warranted to explore whether these cells can be induced into fully mature, long-term surviving, functionally active neurons.

The adult brain harbors neural/glial stem cells in specialized niches [38]. Under physiological conditions, adult neurogenesis occurs mainly in two regions: the subventricular zone and the subgranular zone of the dentate gyrus of the hippocampus [39]. Stem cells in these regions share phenotypical features of radial glia cells or astrocytes expressing GFAP or GLAST [40], [41]. However, neural stem cells have been derived from a variety of other adult brain regions, yet it remains unclear whether these are similar to the neural stem cells found in the subventricular zone and the subgranular zone [42], [43]. Human neural progenitor cells have been isolated from several, also non-neurogenic regions [44], [45], [46], [47].

The perivascular MSC we isolated differ from those previously described human neural stem cells. First, clonal perivascular MSC from the adult human brain do not express mRNA for neural progenitors such as GFAP, PAX6, Musashi, CD133, SOX1 or NGN2 and do not exhibit early neuronal markers (doublecortin, β-III-tubulin) when proliferating. It is therefore highly unlikely that we have isolated one of the known neuronal or glial human progenitor cell types. Second, the here characterized progenitor population clearly exhibits a perivascular and mesenchymal phenotype. Mesenchymal progenitors locate to the perivascular space, but are not restricted to certain neurogenic brain regions. We could isolate perivascular MSC from biopsies from the ventricular zone and from the neocortex and did not observe any difference in marker expression or differentiation capacity. Third, in contrast to neural stem cells, the isolated perivascular MSC have the capacity for both neuroectodermal and mesodermal differentiation.

The described cerebral perivascular MSC can be efficiently propagated long-term as adherent cultures, they show clonality, have a stable karyotype and retain their full differentiation capacity even following extensive proliferation. Thus, the isolated human cerebral perivascular MSC fulfill the criteria for long-term self-renewing stem cells *in vitro* [40].

In conclusion, we have demonstrated the presence of a previously uncharacterized stem cell *in vivo* in a perivascular location in the human brain. We have shown that these perivascular progenitors share a pericyte and mesenchymal phenotype, that they can be isolated and that single cell-derived clones have the capacity to differentiate along both, mesodermal and neuroectodermal lineages *in vitro*. The *in vivo* function of this stem cell, however, is unknown. Future studies will examine its role in the vascular niche in the normal brain and, e.g., how it responds to pathological conditions.

## Materials and Methods

### Human tissue

All procedures involving human tissue were performed with informed written consent by the patient for the donation of brain tissue and were approved by the ethical committees of the Karolinska Hospital, Stockholm and Scania University Hospital, Lund, Sweden. Brain tissue was harvested from individuals undergoing surgery for ventriculostomy or shunt-placement (n = 2) or surgery for intractable temporal lobe epilepsy (n = 2).

### Immunohistochemistry

Cryosections (15 µm) of small human brain cortex pieces were prepared and analyzed as described for human tissue sections [8].

### Isolation and cell culture

Fresh tissue samples were stored in Leibowitz-15 media (Invitrogen) at 4°C, cut and enzymatically digested in enzyme solution (Collagenase 1 mg/ml (Sigma); Dispase 1.6 mg/ml (Roche); Trypsin 0.25 mg/ml (Sigma); DNase I 80 U/ml (Sigma) in Dulbecco's modified Eagle Media (DMEM) and 4.5 mg/ml glucose (Invitrogen) at 37°C/20 min. Cells were plated on 24-well culture dishes and incubated at 37°C/5% CO_2_ in DMEM/F-12/Glutamax/B27 (Invitrogen). Epidermal growth factor (EGF) 20 ng/ml (BD Biosciences), and human basic fibroblast growth factor (bFGF) 20 ng/ml (Invitrogen) were added the day after initial plating. After 7 days, cells were propagated in Stemline medium (Sigma-Aldrich), 2% FBS (Invitrogen), 1% Penicillin/Streptomycin (P/S) (Gibco) and 1% Glutamax (Gibco). Cells were then expanded on uncoated culture flasks (Corning) without the presence of mitogens.

### FACS

For cell sorting, cells were incubated with anti-CD13-PECY7 (BD), anti-CD105-APC (Invitrogen), anti-CD31-FITC (BD), CD45-PE (BD) at the concentration of 20×10^6^ cells/ml and sorted by FACS (FACSAria; Becton Dickinson) or DIVA software (Becton Dickinson) using a low stream speed to ensure a high level of cell survival and in the four-way purity sorting mode to obtain the highest purity of the sorted cells.

### Flow Cytometry

Cultured cells were labeled at different passages with the following commercial antibodies: anti-CD11b-PE, anti-CD14-FITC, anti-CD29-PE-CY5, anti-CD31-FITC, anti-CD34-PerCP, anti-CD45-FITC, anti-CD56-APC, anti-CD49d-PE, anti-CD90-APC, anti-CD140b-PE, anti-CD146-FITC (all Serotec), anti-CD166-PE, anti-CD105 (Invitrogen), anti-CD13-PECY7 and anti-CD73-PE (BD), anti-α-SMA and anti-O4 (Sigma), anti-NG2 (Chemicon), anti-Nestin (Santa Cruz), anti-GFAP Alexa 647, anti-RGS5 (Invitrogen), anti-Kir6.1 (Abcam). Isotype control immunoglobulins used were IgG1-PE, IgG1-FITC, IgG1-APC, IgG1-PercP, IgG1-PECY7 (all from BD). Goat anti-mouse 488 and goat anti-rabbit Alexa 488 were used for non-conjugated primary antibodies. Resuspended cultured cells were stained as follows: For detection of intracellular antigens, cells were fixed with ice-cold methanol (Merck) and permeabilized with 0.05% Saponin (Sigma) when necessary. After washing the cells twice with PBS containing 1% FBS cells were then incubated with the indicated primary antibodies for 30 min on ice. Cells were washed twice, and 10,000 events were acquired on a FACSCalibur (BD).

### Cloning

Clonal cell lines were established using limited dilution to 150 cells/ml and 10 µl placed into 48 wells that were inspected for the presence of a single cell 1 h later and supplemented with medium to the total volume of 300 µl. To determine the frequency of clones, single cells were plated into ×48 well plates and inspected for presence of single cells as above. Proliferating clones were counted as successful once they could be expanded to a minimum of 250,000 cells.

### Differentiation

For glial differentiation, cells were incubated for 10 days in Neurobasal medium (Nb, Invitrogen), LIF (Leukemia inhibitory factor, 10 ng/ml, Chemicon) and CNTF (Ciliary neurotrophic factor, 10 ng/ml, R&D). For neuronal differentiation, cells were grown in Nb medium containing 20 ng/ml PDGF (platelet-derived growth factor) 300 µM, cAMP, 20 ng/ml SHH (sonic hedge hog) and 5 ng/ml FGF2. Medium was supplemented every 48 hrs.

For adipogenesis, cells were grown in DMEM, 10% rabbit serum, 1 µM dexamethasone, 0.5 µM isobutylmethylxanthine, 60 µM indomethacine, and 170 µM insulin (all Sigma). After 14 days, cells were fixed, washed in 60% isopropanol, and incubated with Oil red O (Sigma) for 10 min at room temperature (RT). For chondrogenesis, 450,000 cells were grown as pellets in serum-free DMEM with insulin-transferrin-selenious (ITS) acid mix (Sigma), 50 µg/ml L-ascorbic acid 2-phosphate (WAKO), 100 µg/ml sodium pyruvate (Invitrogen), 0.1 µM dexamethasone (Sigma), and 10 ng/ml transforming growth factor β3 (TGF-β3; R&D). After 21 days, pellets were fixed, frozen, cut into 5 µm sections and stained with Safranin O Red (Sigma). For osteogenesis, cells were cultivated in RPIM, 10% horse serum (Gibco), 0.1 µM dexamethasone, 50 µg/mL L-ascorbic acid, and 10 mM β-glycerophosphate (Sigma). After 21 days, cells were fixed in ice-cold 70% Ethanol and incubated for 30 min with alizarin red (pH 4.2) for the detection of calcium deposits (all reagents from Sigma-Aldrich) or fixed in 4% PFA and stained with osteocalcin (R&D).

### Immunocytochemistry

For immunocytochemistry, cells were plated on glass coverslips that were coated with Poly-L-lysine 90 µg/ml. Cultured cells were fixed 5 min with ice-cold 50% acetone (Sigma Aldrich) and 50% methanol (Fischer Scientific) or with 2% paraformaldehyde (PFA, Sigma) at RT, washed in PBS and incubated for 1 h in PBS, 5% donkey serum (PAA Laboratories). Cultured cells were then stained for both primary and secondary antibodies in the presence of 0.1% Triton X-100 (Sigma) and 2% donkey serum. Primary antibodies: human-specific anti-Nestin (R&D, Chemicon), anti-GFAP (DAKO), anti-β-III-tubulin (TUJ1) (BioSite), anti-NG2 (Chemicon), anti-α-SMA (Abcam; Sigma), anti-vWF (DAKO), anti-DCX (Chemicon; Cell-Signaling), anti-MAP2 (Sigma), anti-CD31 (DAKO, BD), anti-Synaptophysin (kindly provided by R. Jahn), anti-O4 (Sigma), anti-GABAA-receptor (Millipore); anti-TH (Millipore), anti-Serotonin (Chemicon), Anti-ChAT (Chemicon), anti-CD105 (BD), anti-CD13 (BD), anti-Ki67 (DAKO), anti-PDGFR-β (Cell signaling), anti-RGS5 (Invitrogen), anti-Kir6.1 (Abcam), anti-A2B5 (Chemicon), anti-S100β (Sigma) and anti-PSD 95 (Abcam).

Secondary antibodies: horse-anti-mouse FITC (Vector), donkey-anti-rabbit CY3 (Jackson Immuno Research), donkey-anti-goat FITC (Jackson Immuno Research), goat-anti-mouse IgM FITC (Jackson Immuno Research), biotinylated anti-sheep (Vector laboratories); biotinylated anti-rabbit (Vector laboratories), goat anti-mouse Alexa Fluor 488, goat anti-mouse Alexa Fluor 594, goat anti-rabbit Alexa Fluor 488 and Alexa Fluor 594 and donkey anti-mouse Texas red (Jackson ImmunoResearch Laboratories) were used.

### PCR

For real-time qPCR, five-hundred nanograms of total RNA from each sample were used for Oligo(dT)_16_ reverse transcription using the AmpliTaq Gold® DNA polymerase first-strand synthesis system for RT-PCR (Applied Biosystems). qPCR reactions with synthesized cDNAs were performed on ABI Prism 7100 (Applied Biosystems) using SYBR Green QPCR Master Mix (Fermentas). mRNA levels were normalized using large ribosomal protein as an internal control. Primer sequences are provided on request.

### Karyotyping

Cells from several donors were karyotyped at early, medium and late passage. Cells plated in t25 flasks were harvested using 0.05% trypsin (Sigma, Sweden), and G-banding of chromosomes was obtained with Wright's stain, according to standard procedures. The clonality criteria and the description of the karyotype were according to the recommendations of the ISCN 1995. For each karyotype analyzed, seven different metaphase spreads were examined.

### Image processing and cell counts

Images were acquired using either Olympus BX60, Olympus IX70 or Leica DMRBE fluorescence microscopes. Images were captured with digital cameras (Olympus DP50 and Hamamatsu C4742-95) and processed using studio and Openlab softwares. Confocal microscopy was performed using a Leica microscope equipped with a GreNe and a HeNe laser, using the following lines of excitation: 488 nm, 594 nm and 647 nm. Figures were composed using CANVAS software. Cells were counted in triplicate cultures in randomly chosen areas under the microscope. A minimum of 100 cells/experiment was assessed. Cell numbers are expressed as mean standard deviation (SD).
